# Associations of Fetuin-A levels with vascular disease in type 2 diabetes patients with early diabetic nephropathy

**DOI:** 10.1186/1475-2840-9-48

**Published:** 2010-09-07

**Authors:** Marcel Roos, Dimitrios Oikonomou, Maximilian von Eynatten, Peter B Luppa, Uwe Heemann, Jens Lutz, Marcus Baumann, Peter P Nawroth, Angelika Bierhaus, Per M Humpert

**Affiliations:** 1 Department of Nephrology, Klinikum rechts der Isar, Munich, Germany; 2 Department of Clinical Chemistry and Pathobiochemistry, Klinikum rechts der Isar, Munich, Germany; 3 Department of Medicine I and Clinical Chemistry, University of Heidelberg, Heidelberg, Germany

## Abstract

**Background:**

Ambigous results exist on fetuin-A as marker for vascular disease in type 2 diabetes. This study aims to define the role of fetuin-A as marker for micro- and macrovascular disease in a high risk population of patients with type 2 diabetes mellitus and early diabetic nephropathy.

**Methods:**

Fetuin-A serum levels were assessed by ELISA in a cross-sectional setting in 153 patients with type 2 diabetes. Associations of fetuin-A with metabolic, inflammatory and vascular markers were studied. Atherosclerotic burden was assessed by ankle-brachial-index (ABI) as well as detection of common carotid artery intima-media thickness (IMT).

**Results:**

Levels of fetuin-A were lower in male than in female patients (0.49 ± 0.15 vs. 0.56 ± 0.20 g/L, p = 0.02). In addition, there was an inverse correlation with age (r = -0.20, P = 0.01). Bivariate correlations adjusted for age and gender revealed no significant correlations with metabolic parameters, except for a weak inverse correlation with serum adiponectin (r = -0.19, p = 0.02). Regarding parameters of micro- and macrovascular disease, fetuin-A was significantly associated with ABI (r = 0.18, p = 0.04), while there was no association with IMT (r = -0.07, p = n.s). Patients with an ABI < 0.9 had lower fetuin A levels than patients with an ABI 0.9-1.3 or > 1.3 (0.43 ± 0.10 vs. 0.52 ± 0.17 vs. 0.54 ± 0.18 g/L p = 0.05). Neither GFR nor albuminuria were associated with fetuin-A serum levels. Patients with prevalent neuropathy did not have altered fetuin-A levels compared to diabetic controls. In step-wise logistic regression analysis including age, gender, HbA1c, total cholesterol, glomerular filtration rate and fetuin-A, only total cholesterol (β = 0.01, p = 0.02) and fetuin-A (β = -5.99, p = 0.03) proved to be independent predictors of an ABI < 0.9.

**Conclusions:**

The results of this cross-sectional study suggest that lower fetuin-A levels are associated with macrovascular late complications in high-risk type 2 diabetes patients while there are no associations of fetuin-A with metabolic status or microvascular complications.

## Background

Various biomarkers have been studied for identification of type 2 diabetes (T2D) patients at micro- and macrovascular risk. Most of these markers are inflammatory, metabolic or procoagulant molecules indicating an unfavourable metabolic and vascular status in patients with type 2 diabetes [1]. However, the different biomarkers show large variations in risk prediction depending on metabolic status and disease severity of the study groups [2,3]. Recently published data imply, that most novel biomarkers do not improve risk prediction when added to models based on conventional risk scores [4] Yet, associations of novel biomarkers such as fetuin-A with metabolic markers or complications do help to understand their role in the pathophysiology of vascular disease.

In mice, fed a mineral/vitamin D-rich diet, knockout of fetuin-A resulted in arterial or soft-tissue calcification or both [5-7]. In humans, so far the available data has been inconsistent. Lower fetuin-A levels are associated with mortality and CVD events in cohorts with end-stage renal disease (ESRD) [8,9], while a population based study linked high plasma fetuin-A levels to an increased risk of myocardial infarction (MI) and ischemic stroke (IS) [10]. Yet, we could not demonstrate an association of fetuin-A with traditional cardiovascular risk factors, cardiovascular outcome or the metabolic syndrome in patients with manifest CHD in a 6-year follow study [11]. Contradictory results have also been published regarding the role of fetuin-A in macrovascular disease and patients with type 2 diabetes [12-14]. While some studies associated lower fetuin-A levels with peripheral arterial disease (PAD) [12,13], others observed an association of increased fetuin-A levels with coronary artery calcification (CAC) [15].

So far, there is no data available for associations of fetuin-A with parameters of microvascular dis-ease in diabetes. To clarify the relation between fetuin-A and microvascular complications in patients with type 2 diabetes and early diabetic nephropathy, we studied associations with albuminuria, parameters of renal function as well as diabetic neuropathy. In addition, the atherosclerotic burden of these patients was assessed by quantification of the ankle-brachial index (ABI) and the common carotid artery intima-media-thickness (IMT).

## Methods

### Study population and data collection

153 Patients with type 2 diabetes were recruited from family practices being referred to the diabetes outpatient clinic at the University Hospital Heidelberg for specialist treatment. For eligibility, patients had to have a documented history of albuminuria in at least two separate urine samples (urinary albumin > 20 mg/L as suggested by current German and international guidelines for the diagnosis of diabetic nephropathy [16] as previously described [17]. Detailed patient characteristics are given in Table 1. In all individuals, 24-h urine samples were collected on 3 consecutive days and the mean of AER as well as the MDRD formula for the estimation of the glomerular filtration rate [18] were taken for statistical evaluation of renal function. All blood values, as well as ambulatory 24-h blood pressure values (given as mean of 24 h), were taken on day 1. The study complied with the Declaration of Helsinki, and all subjects gave written informed consent. The study was approved by the ethics committee of the Uni-versities of Heidelberg.

**Table 1:** Patients Characteristics

	Type 2 diabetic patients	Serum fetuin-A*	
		
		r	p
n	153		

Gender (male)	116 (74)§	-	-

Age (Years)	59 ± 8	-0.201#	0.01

BMI (kg/m^2^)	33 ± 6	-0.125	n.s.

Systolic BP (mmHg)	136 ± 19	0.104	n.s.

Diastolic BP (mmHg)	80 ± 8	0.125	n.s.

Duration of diabetes (years)	12 ± 8	-0.016	n.s.

Current smoker (yes)	25 (16)	-	-

Patients on ACE-inhibitor or angiotensin receptor blocker (yes: n;%)	128 (82)	-	-

Patients on Statin (yes:n;%)	89 (57)	-	-

Patients on Beta-Blockers (yes:n;%)	76 (49)	-	-

Patients receiving antihypertensive drugs (%)	137 (90)	-	-

Patients receiving > 2 antihypertensive drugs (%)	73 (48)	-	-

Patients on Insulin Treatment (yes:n;%)	91 (58)	-	-

HbA1c (%)	7.3 ± 1.2	-0.045	n.s.

Fasting Glucose (mg/dL)	144 ± 50	-0.022	n.s.

Serum Creatinine (mg/dL)	0.89 ± 0.3	0.017	n.s.

Serum Albumin (g/L)	45 ± 3	-0.006	n.s.

Serum Fetuin A (g/L)	0.51 ± 0.17		

* log transformed data for analysis

# adjusted for gender, all other correlations are partially adjusted for age and gender

§ male vs. female: 0.49 ± 0.15 vs. 0.56 ± 0.20 g/L, p = 0.02

### Clinical chemistry

Blood was drawn on day 1 in a fasting state under standardized conditions and stored at -80°C until analysis. Serum fetuin-A was measured in duplicates by an ELISA (Epitope Diagnostics, Inc., San Diego, USA) according to the manufacturer's protocol. The intra- and interassay variations were 5.3 and 7.1%, respectively. FPG was measured by a glucose oxidase method. Triglyceride, total cholesterol, and HDL cholesterol levels were quantified by standard laboratory methods, and LDL cholesterol levels were calculated by using the Friedewald formula. A1C was measured by high-performance liquid chromatography on a Variant II device (Bio-Rad Laboratories, Munich, Germany). AER was assessed and performed in three consecutive 24-h urinary collections. For collection of the urine sample, a 3-l plastic container was used, and the volume of urine was measured to the nearest 50 ml. Albumin levels were determined by turbidimetry (Siemens Healthcare Diagnostics, Eschborn, Germany). AER was expressed as milligrams per 24 h.

### Assessment of carotid atherosclerosis and ankle-brachial index

IMT was detected using high-resolution B-mode ultrasound (Voluson 730 Kretz, Tiefenbach, Austria) of the extracranial carotid arteries bilaterally under continuous detection of the heart cycle using a three-lead electrocardiogram. The whole imaging and quantification procedure was performed digitally (Voluson 730 Kretz, Tiefenbach, Austria) at the time of study entry by a single investigator blinded for clinical data. For the purpose of this study, IMTs of the far wall of the common carotid artery were detected in end-diastolic frames, ~10 mm proximal to the carotid bulb, according to a previously described scanning protocol [19]. The measurements were performed at four points of both common carotid arteries avoiding areas of atherosclerotic plaque formation. The mean of the resulting eight single measurements was taken as mean IMT for statistical analyses in this study. The ABI was detected by simultaneous measurement of the brachial and posterior tibial artery systolic pressure using the 5 MHz Mini Dopplex^® ^doppler device (Huntleigh Diagnostic Products, Cardiff, UK). Pressure was taken at the posterior tibial artery only and the lower ABI from both sides was taken for analyses. Patients were further classified to have no PAD (ABI 0.9-1.3), mediasclerosis (ABI > 1.3) or prevalent PAD (ABI < 0.9). 151 patients qualified for statistical analyses regarding this endpoint.

Patients were considered to have significant neuropathy when they had a vibration perception < 5/8 using a Rydel-Seiffer graduated tuning fork (C-64 to C-128) which was previously shown to be a reliable diagnostic instrument for the detection of peripheral neuropathy [20]. The mean of measurements on both feet was taken for evaluation.

### Statistical analyses

Statistical analyses were performed using SPSS software version 16.0 (SPSS, Chicago, IL). Spearman correlation coefficients were used to describe the association between serum fetuin-A and the variables of interest. Comparison between two or more sets of patients was performed by independent t test or one-way ANOVA. Multivariable linear and stepwise binary logistic regression analyses evaluated the association of serum fetuin-A with the respective metabolic or vascular parameters. Models were adjusted as given in the respective Table or the Results section.

## Results

### Association of serum fetuin-A levels with cardiovascular risk factors and renal function

Clinical and laboratory characteristics of patients with type 2 diabetes and bivariate analyses of as-sociations between different variables and serum fetuin-A are given in Table 1. The overall mean serum level of fetuin-A was 0.51 ± 0.17 g/L with a range of 0.30 to 1.07 g/L. In bivariate analysis, only age was significantly and inversely associated with serum fetuin-A levels (r = -0.20, p = 0.02). Male patients had significantly lower fetuin-A levels (0.49 ± 0.15 vs. 0.56 ± 0.20 g/L, p = 0.02). Table 2 shows the association of fetuin-A levels with metabolic pa-rameters and markers of vascular disease and renal function. Apart from adiponectin, which was inversely and signifi-cantly associated with fetuin-A levels (r = -0.188, p = 0.02), no other metabolic variable significantly correlated in this group of type 2 diabetes patients with vascular late complications.

**Table 2:** Associations of Fetuin A with Metabolic Parameters, markers of vascular disease and renal function

		Serum fetuin-A*	
		
		r#	p
Waist/Hip-Ratio	1.02 ± 0.08	0.069	n.s.

Triglycerides (mg/dL) *	223 ± 307	0.05	n.s.

Cholesterol (mg/dL)	189 ± 56	-0.001	n.s.

HDL (mg/dL)	45.6 ± 13.4	-0.034	n.s.

Adiponectin (μg/mL) *	10.3 ± 6.6	-0.188	0.02

hsCRP (mg/L)*	3.9 ± 5.4	0.063	n.s.

Intima-Media-Thickness (mm)	0.87 ± 0.15	-0.070	n.s.

Ankle-Brachial Index	1.01 ± 0.18	0.175	0.04

Estimated Glomerular Filtration Rate (MDRD, ml/min)	97 ± 32	-0.017	n.s.

Albumin Excretion (mg/24 h)*	154 ± 371	0.096	n.s.

* log transformed data for analysis

# partially adjusted for age and gender

We could not identify a significant correlation of serum fetuin-A levels with IMT, the glomerular filtration rate (GFR by MDRD) or the albumin excretion. However, serum fetuin-A was positively associated with the ABI (r = 0.18, p = 0.04). In multivariable linear regression analysis with fetuin-A as the dependent variable, age (β = -0.203, p = 0.024) and adiponectin (β = -0.192, p = 0.03) were independently associated with fetuin-A. A boderline significance was seen with gender (β = 0.228, p = 0.06). No associations of fetuin-A were seen with BMI (β = -0.131, p = 0.12), HbA1c (β = -0.004, p = 0.96) and albumin (β = -0.02, P = 0.98).

**Figure 1:**
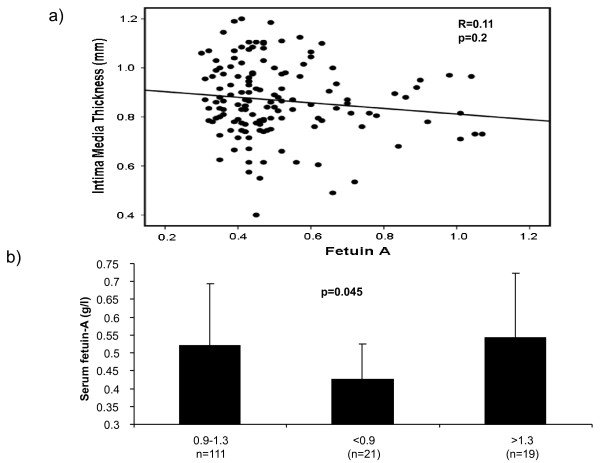
**A: Serum fetuin-A levels and macrovascular disease (Intima Media Thickness)**. Scatter plot showing the relationship between serum fetuin-A levels and IMT. Data as given by Spearman correlation coefficient. **B: Serum fetuin-A levels and ankle-brachial index (ABI)**. Fetuin-A levels were compared between groups stratified by ABI.(< 0.90, 0.90-1.30, and > 1.30). Data +/-SD, p-value as given by one- way ANOVA.

**Figure 2:**
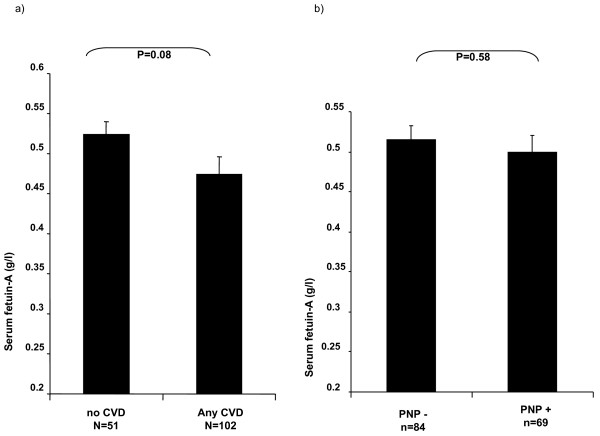
**A: Serum fetuin-A levels and cardiovascular disease (CVD)**. Patients were stratified by history of any cardiovascular disease (i.e. PAD, CAD or stroke). Data +/-SEM, p-value as given by independent T-Test. **B: Serum fetuin-A levels in patients with diabetic neuropathy**. Data +/-SEM, p-value as given by independent T-Test.

Associations of fetuin-A levels with prevalent macrovascular disease were studied. While there were no associations of fetuin-A with IMT as a surrogate marker of atherosclerosis (r = -0.11, p = 0.2, Figure 1a), patients with an ABI < 0.9 had lower fetuin A levels than patients with an ABI 0.9-1.3 or > 1.3 (0.43 ± 0.10 vs. 0.52 ± 0.17 vs. 0.54 ± 0.18 g/L p = 0.05). (Figure 1b). In addition, concentrations of fetuin-A were quantified in patients with or without a history of macrovascular disease such as coronary artery disease, stroke and peripheral artery disease. In this analysis, patients with a history of macrovascular disease (n = 102) revealed a trend towards lower levels of fetuin-A compared to patients without prevalent macrovascular disease (n = 51) (0.47 ± 0.16 vs 0.52 ± 0.17 g/L, p = 0.08), (Figure 2a). In a binary stepwise logistic regression analysis excluding patients with an ABI > 1.3, only total cholesterol (β = 0.01, p = 0.02) and fetuin-A (β = -5.99, p = 0.03) proved to be independent predictors of an ABI < 0.9, while age, gender, HbA1c, and glomerular filtration rate were not sig-nificantly associated with PAD.

We further investigated the association of fetuin-A levels with prevalent polyneuropathy (PNP). Fe-tuin-A serum levels did not differ among patients with (n = 69) vs. without (n = 84) PNP (0.50 ± 0.15 vs. 0.52 ± 0.16 μg/mL, p = 0.58) (Figure 2b). Likewise, fetuin-A concentrations were not associated with the degree of 24 h albumin excretion as an additional marker of microvascular disease in these patients with early diabetic nephropathy (Table 2).

## Discussion

This study demonstrates that lower fetuin-A levels seem to be associated with prevalent macrovas-cular disease in type 2 diabetes, but not with IMT as a surrogate marker of atherosclerotic burden. Furthermore, we could show for the first time, that fetuin-A serum levels are not associated with microvascular complications in patients with early diabetic nephropathy. In addition, fetuin-A levels do not correlate with metabolic parameters in our type 2 diabetes patients with prevalent late complications.

Fetuin-A was shown to act as a natural inhibitor of the insulin receptor tyrosine kinase in liver and skeletal muscle, and fetuin knock-out mice displayed improved insulin sensitivity [21]. Different observational studies have proposed that high serum fetuin-A levels are associated with the presence or development of the metabolic syndrome suggesting fetuin-A as a risk factor for this condition [10,22,23]. Moreoever, fetuin-A complexes with calcium and phosphorus in the circulation and prevents the precipitation of these minerals in serum [5,6]. Fetuin-A is regarded as marker for vascular inflammation and as one of the most potent negative regulators of vascular ossification - calcification [24,25]. In animals lacking the fetuin-A gene, the aorta was found to be spared of cal-cification and fibrosis, whereas peripheral vessels in the skin and kidney showed evidence of extensive calcification, and the small artery involvement preceded the impairment of renal function [6,26]. The data presented herein allows to speculate, that fetuin-A could play a role in the development of prevalent macrovascular disease in type 2 diabetes, yet possible mechanisms remain unclear. In line with this hypothesis, Emoto et al. showed a possible effect of fetuin-A on inhibiting the calcification of atherosclerotic calcified plaques (CP) independently of renal impairment [13]. In their study including less advanced type 2 diabetes patients without nephropathy and of which 32% were even on dietary treatment, fetuin-A levels were significantly lower in patients with atherosclerotic CP of the common carotid and femoral arteries than in those without CP. Our data in more advanced micro- and macrovascular disease suggests that fetuin-A might play a role in the development of vascular stenosis, while there were no associations with early lesions indicated by IMT. It needs to be emphasized in this context, that the measurement of IMT is performed in areas with no atherosclerotic plaque. However, patients in this study have a number of possible confounders for the association of fetuin-A and IMT like medications including insulin as well as prevalent microvascular disease. The results presented are also in line with previous data [12] showing that circulating fetuin-A was lower in 38 subjects with type 2 diabetes and an ABI < 0.9, compared with 700 diabetes controls. Somehow contradictory, another study showed a direct relation between serum fetuin-A levels and coronary artery calcification (CAC) evaluated by electron beam computed tomography [15]. Like IMT, CAC is a surrogate marker for coronary artery disease not specifying for the severity of CHD in terms of vascular stenosis. In addition, the data on CAC were obtained from African Americans and Latinos and fetuin-A levels were previously shown to differ among ethnic groups [22,27]. Reviewing the existing data as well as the results obtained in this study, it may be assumed that fetuin-A levels are lower in type 2 diabetes patients with stenosing arterial disease, possibly independent of the localization.

Our results are in contrast to findings in non- diabetic subjects [28], as well as several cross-sectional studies which showed an association of high fetuin-A levels with impaired glucose tolerance, lipid metabolism and markers of vascular disease [10,22,23]. The advanced type 2 diabetes patients in this study showed no associations of metabolic parameters with fetuin-A except and inverse correlation of fetuin-A levels with serum adiponectin which is in line with previous experimental data showing a decrease in adiponectin expression as well as circulating adiponectin levels in mice treated with fetuin-A [21]. Yet, associations of fetuin-A with metabolic parameters can be confounded by many of the medications in the subjects studied herein. In a middle-aged non- diabetic population of which the majority was female, increased fetuin-A levels were associated with increased IMT [28]. Again, a recently published study in patients without diabetes and renal impairment demonstrated decreased fetuin-A serum levels in patients suffering from advanced three-vessel disease compared with those without stenosis [29]. These finding go in line with our assumption of reduced fetuin-A serum levels in patients with prevalent vascular disease.

Taken together, we assume a biphasic association of fetuin-A with vascular disease: relatively healthy diabetic and non-diabetic patients without pre-existing vascular disease [10,23] show associations of higher fetuin-A with metabolic and vascular risk, while patients with prevalent vascular disease have decreased fetuin-A levels. Hence, the currently available data implies that effects of fetuin-A may be of greater importance in the very early phases of atherosclerosis, a pattern known from other CHD risk factors, such as C-reactive protein or adiponectin, which seem to be better predictors in primary prevention [30,31]. Yet, future experimental studies will have to reveal the mechanisms by which fetuin-A could influence the early atherosclerotic process.

Since mice deficient for the fetuin-A gene showed a distinct pattern of small caliber vessel and kidney calcificiation, we studied associations of parameters of microvascular disease with fetuin-A for the first time in this study. Interestingly, we did not find any associations, neither with the degree of albumin excretion or renal function, nor with the prevalance of diabetic neuropathy. This suggests, that fetuin-A does not play a significant role in the development of these entities of diabetes complications.

We recognize the limitations of the present study. Our approach is limited by the small samples size. Therefore, especially the data for associations with microvascular disease and renal function in type 2 diabetes will have to be reproduced in larger groups. Moreover, the cross-sectional design needs cautious interpretation of data and there is no prospective data for primary or secondary prevention in diabetes available.

## Conclusions

In this study, low plasma fetuin-A levels predict PAD in type 2 diabetes with early diabetic neph-ropathy while there are no associations of fetuin-A with markers of microvascular disease in these patients.

## Competing interests

The authors declare that they have no competing interests.

## Authors' contributions

All authors participated in the design and coordination of the study, reviewed the analysis and took part in writing the manuscript. They also read and approved the final manuscript.
